# Fracture Risk After Bariatric Surgery: A 12-Year Nationwide Cohort Study

**DOI:** 10.1097/MD.0000000000002087

**Published:** 2015-12-07

**Authors:** Chia-Wen Lu, Yu-Kang Chang, Hao-Hsiang Chang, Chia-Sheng Kuo, Chi-Ting Huang, Chih-Cheng Hsu, Kuo-Chin Huang

**Affiliations:** From the Department of Family Medicine, National Taiwan University Hospital, Taipei (C-WL, H-HC, C-SK, K-CH); Institute of Population Health Sciences, National Health Research Institutes, Zhunan, Miaoli County (Y-KC, C-TH, C-CH, K-CH); Department of Family Medicine, College of Medicine, National Taiwan University, Taipei (K-CH); Department of Health Services Administration, China Medical University, Taichung City (C-CH); and Institute of Clinical Medicine, National Yang Ming University, Taipei, Taiwan (C-CH).

## Abstract

Bariatric surgery has been shown to impair bone health. This study aimed to investigate the fracture risk in patients after bariatric surgery versus propensity score-matched controls. The authors used the National Health Insurance Research Database of Taiwan and identified 2064 patients who underwent bariatric surgery during 2001 to 2009. These patients were matched to 5027 obese patients who did not receive bariatric surgery, using propensity score matching accounting for age, sex, Charlson Comorbidity Index, diabetes, hypertension, hyperlipidemia and the year morbid obesity was diagnosed. The authors followed the surgical and control cohorts to death, any diagnosis of fracture, or December 31, 2012, whichever occurred first. Cox proportional hazard regression models were used to calculate relative rates of fractures in the surgical group and control group. At the end of the 12-year study period, there were 183 fractures in the surgical group (mean follow-up 4.8 years) and 374 fractures in the matched control group (mean follow-up 4.9 years). Overall, there was a 1.21-fold [95% confidence interval (CI): 1.02–1.43] significantly increased risk of fracture in the surgical group compared with the control group. Stratified by surgical procedures, malabsorptive procedures showed a significantly higher fracture risk (1.47, 95% CI: 1.01–2.15). The Kaplan-Meier estimated fracture rates were 1.60% at 1 year, 2.37% at 2 years, 1.69% at 5 years, and 2.06% after 5 years for the surgical patients, compared with 1.51%, 1.65%, 1.53%, and 1.42%, respectively, for the matched controls. Adjusted analysis showed a trend towards an increased fracture risk, 1 to 2 years after bariatric surgery. (1.42, 95% CI: 0.99–2.05). Bariatric surgery was significantly associated with an increased risk of fractures, mainly with malabsorptive procedures, with a trend of an increased fracture risk 1 to 2 years after surgery. These results provide further evidence for the adverse effects of bariatric surgery on the risk of fractures.

## INTRODUCTION

Obesity is an important and increasing public health issue worldwide. The prevalence of morbid obesity in the United States is more than 5% of the population,^1^ and the estimated prevalence of obesity in Europe is 15% to 20% among the middle-aged.^2^ As the most effective treatment for obesity in achieving significant and durable weight loss, the use of bariatric surgery has increased at least 7-fold worldwide in the last decade.^3^ The long-term survival and potential effects on health after bariatric surgery, however, are not clearly understood.^4^ There is accumulating observational evidence that bariatric surgery is associated with a negative effect on bone health, such as elevated bone turnover markers and reduced bone mineral density (BMD), thereby accelerating bone loss and increasing skeletal fragility.^5–8^

Several prospective studies have explored the relationship between bariatric surgery (grouped as restrictive or malabsorptive types) and changes in BMD, and reported that patients who undergo bariatric surgery suffer a decrease in BMD, and that this effect is more significant with malabsorptive procedures.^8–10^ The results, however, have been inconsistent with regards to the affected bone regions^11,12^ and time course.^13–16^ In addition, little is known about the incidence of osteoporosis after bariatric surgery.^17^ To date, only 2 large retrospective studies have reported an association between bariatric surgery and fracture risk. In Olmsted County in the United States, patients receiving bariatric surgery seemed to be associated with an increased fracture risk^18^ whereas a retrospective study conducted in England showed no association between bariatric surgery and the risk of fractures.^19^

In light of these controversial findings, we conducted this nationwide 12-year follow-up cohort study with the hypothesis that bariatric surgery would increase the fracture risk. We also aimed to determine the relationship between bariatric surgery and the risk of fractures among different observation periods, surgical types, and bone regions.

## MATERIALS AND METHODS

### Data Source and Study Subjects

The data source of this study was based on the National Health Insurance Research Database (NHIRD) which is derived from the reimbursement claim records of the National Health Insurance (NHI) program, a universal single-payer health insurance program implemented in Taiwan in 1995. The NHI Administration has contracts with more than 95% of healthcare facilities, and over 99% of the 23 million Taiwanese population has now registered in the NHI program,^20^ indicating that the NHI program is the most important component of the healthcare system in Taiwan. The National Health Insurance Research Database includes detailed information about the medical utilization of NHI beneficiaries, including date of birth, sex, residential area, medical diagnostic codes based on the International Classification of Diseases, Ninth Revision, Clinical Modification (ICD-9-CM), drug prescriptions and medical procedures. The study has been approved by the institutional review board of National Health Research Institutes in Taiwan (EC1030701-E).

The patients with prevalent morbid obesity (ICD-9-CM 278.01, n = 21,179) diagnosed between 2001 and 2009 were divided into 2 groups based on whether or not they received bariatric surgery after the first diagnosis of morbid obesity during the follow-up period. For the recipients who underwent bariatric surgery (n = 2707), we excluded those who had a prior diagnosis of a fracture (ICD-9-CM 800–829) or osteoporosis (ICD-9-CM 733.0) before surgery (n = 471) and those with missing data (n = 1), and the remaining 2064 subjects were designated as the surgical group for further analysis. The date of bariatric surgery was defined as the index date. The principal ICD-9-CM procedure codes for bariatric surgery were 43.82 (laparoscopic sleeve gastrectomy), 43.89 (open and other partial gastrectomy), 44.31 (high gastric bypass), 44.38 (laproscopic gastroenterostomy), 44.39 (other gastroenterostomy without gastrectomy, 44.68 (laparoscopic gastroplasty), 44.69 (other repair of stomach), 44.95 (laparoscopic gastric restrictive procedure), and 44.99 (other operations on stomach). The surgical cohort was further classified into malabsorptive procedures (ICD-9-CM 44.31, 44.38, and 44.39) and restrictive procedures (ICD-9-CM 43.82, 43.89, 44.68, 44.69, 44.95, and 44.99).

For those who did not receive bariatric surgery (n = 18,472), we used propensity score matching to increase their comparability to the surgical group.^21^ We estimated the propensity score for each obese patient using nonparsimonious multivariate logistic regression, with undergoing bariatric surgery as the dependent variable. We incorporated clinically important covariates, including age, sex, Charlson Comorbidity Index (CCI), diabetes, hypertension, hyperlipidemia, and the year that morbid obesity was diagnosed as independent variables. We defined the first record of morbid obesity in the database as the year of diagnosis for patients who had longstanding obesity. The nearest neighbor algorithm was applied to construct the surgical and nonsurgical matched pairs at a 1:3 ratio, assuming that a proportion of 0.995 to 1.0 was perfect.^22^ We then assigned the corresponding index date of each individual in the surgical group to their respective nonsurgical matched counterparts (n = 6192). Those who withdrew from the NHI program (n = 109) or had diagnosis of fracture or osteoporosis before the assigned index date (n = 1056) were also excluded. The remaining 5027 propensity score-matched subjects were designated as the control group for further analysis. We followed both the surgical and control cohorts to death, any diagnosis of fracture, or December 31, 2012, whichever occurred first.

### Definition of Research Variables

The main outcome of the current study was the occurrence of any fracture, defined as the main admission diagnosis during the follow-up period. We further classified the types of fracture according to the following sites: skull/face (ICD-9-CM 800–804), hands/fingers (ICD-9-CM 815–817), distal forearm (ICD-9-CM 813–814), proximal humerus (ICD-9-CM 812), clavicle/scapula/sternum (ICD-9-CM 807.2–807.3, and 810–811), ribs (ICD-9-CM 807.0–807.1), thoracic/lumbar vertebrae (ICD-9-CM 805.2–805.5 and 806.2–806.5), cervical vertebrae (ICD-9-CM 805.0–805.1 and 806.0–806.1), pelvis (ICD-9-CM 808), proximal leg (ICD-9-CM 820–821), distal leg (ICD-9-CM 822–824), and feet/toes (ICD-9-CM 825–826).

A history of diabetes (ICD-9-CM code 250), hypertension (ICD-9-CM code 401), or hyperlipidemia (ICD-9-CM code 272) was defined as when the study subjects had at least 1 hospitalization or 2 ambulatory visits with the respective diagnosis within 3 years before the first diagnosis of obesity. We also estimated the CCI score as the comorbid condition of each study subject.^23^

### Statistical Analysis

The baseline characteristics of the study population are presented as the frequency with percentage for categorical variables and the mean with standard deviation for continuous variables. Differences between the surgical and control groups were compared by *t* tests and χ^2^ tests where appropriate. The incidence rate of fractures was calculated as the number of events (fractures) divided by the number of person-years in the observation period. For each study subject, person-years were calculated as the time elapsed from the assigned index date until the individual died, had a fracture, or reached the end of follow-up (December 31, 2012). Cox proportional hazards regression was used to estimate the adjusted hazard ratios (aHRs) and 95% confidence intervals (CIs) of fractures in the surgical group compared with the controls. Proportional hazards assumption was assessed using the Schoenfeld residuals test and complementary log–log plots. For the multivariate analyses, we adjusted for all covariates listed in Table 1. To compare the effects of different surgical procedures, we conducted subgroup analysis by dividing the surgical group into 2 subgroups: those who received malabsorptive procedures and those with restrictive procedures. To observe changes in fracture risk across the follow-up period, we also assessed the HRs of fracture in the following time periods: ≤3, 4–12, 13–24, 25–60, and >60 months after the index date.

**TABLE 1:**
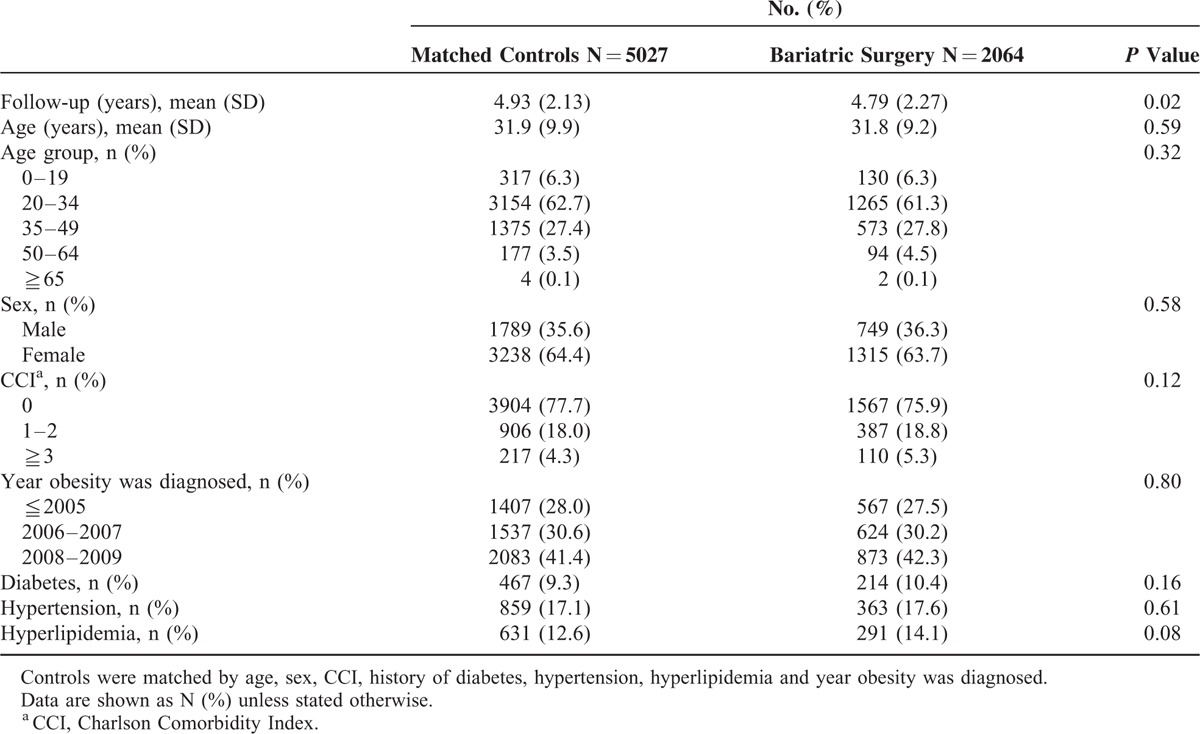
Baseline Characteristics of the Patients Receiving Bariatric Surgery and the Matched Controls

In all analyses, a 2-sided *P* value of less than 0.05 was considered to indicate statistical significance. All analyses were performed using SAS software version 9.3 (SAS Institute Inc., Cary, NC).

## RESULTS

Table 1 shows the baseline characteristics of the surgical and control groups. The surgical patients had a mean age of 31.8 years and 63.7% were women. The matched control patients had a mean age of 31.9 years and 64.4% were women. The 2 groups were similar in most of the baseline characteristics on which they were matched.

Comparisons of the overall risk of fractures between the 2 groups are shown in Table 2. At the end of the 12-year study period, there were a total of 183 fractures in the surgical group (mean follow-up 4.8 years, 1.84 cases per 1000 person years) and 374 fractures in the matched control group (mean follow-up 4.9 years, 1.50 cases per 1000 person years). Overall, there was a 1.22-fold (95% CI: 1.02–1.45) significantly increased risk of fractures in the surgical group compared with the controls. After adjusting for age, sex, CCI, diabetes, hypertension, hyperlipidemia, and the year obesity was diagnosed, there was still a significantly higher fracture rate in the surgical group (aHR: 1.21, 95% CI: 1.01–1.44) compared with the controls. With regards to the type of surgery, 289 (14%) subjects underwent malabsorptive procedures and 1775 (86%) underwent restrictive procedures. There were 29 fractures in the malabsorptive group and 154 fractures in the restrictive group. We observed an increase in the adjusted relative risk for malabsorptive (aHR: 1.47, 95% CI: 1.01–2.15) but not for restrictive (aHR: 1.17, 95% CI: 0.97–1.41) procedures compared with the controls.

**TABLE 2:**
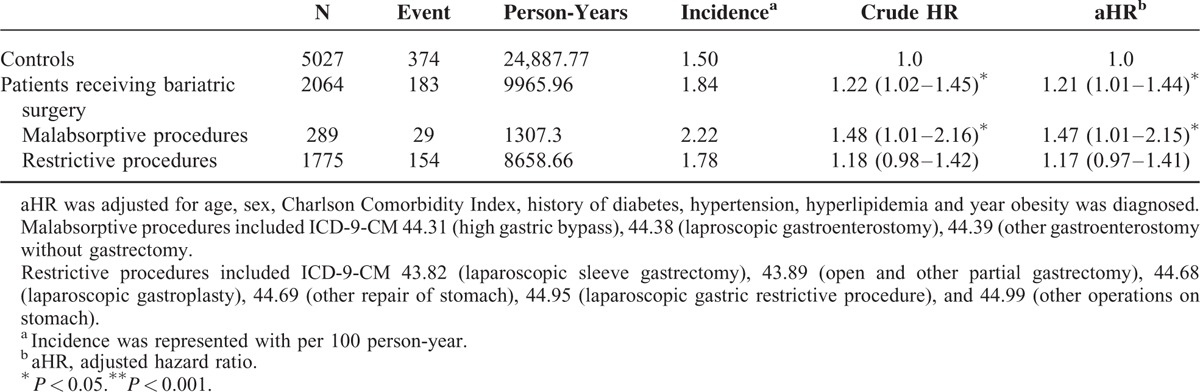
Risk of Fractures in the Patients Receiving Bariatric Surgery Compared With the Matched Controls, by Type of Surgical Procedure

**FIGURE 1:**
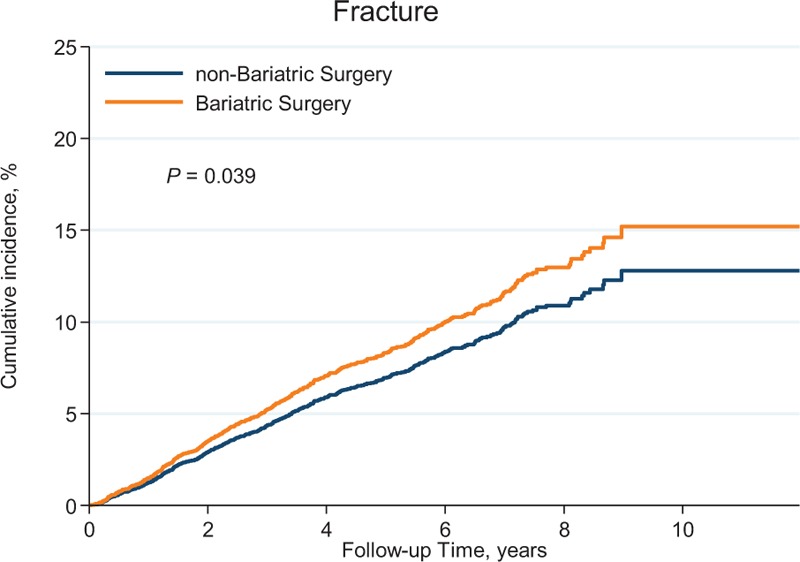
Cumulative incidence of fracture in the patients receiving bariatric surgery and the matched controls.

In Kaplan-Meier analysis (Table 3 and Fig. 1), the fracture rates were 1.60% at 1 year, 2.37% at 2 years, 1.69% at 5 years, and 2.06% after 5 years for the surgical group, compared with 1.51%, 1.65%, 1.53%, and 1.42%, respectively, for the matched controls. Adjusted analysis showed a trend toward an increased overall risk of fractures 1 to 2 years after bariatric surgery, however, this did not achieve statistical significance (aHR: 1.42, 95% CI: 0.99–2.05). In addition, our findings revealed a reduction in the fracture risk in the surgical group after 2 years, and then a trend toward an increased fracture risk after 5 years, although neither of these trends achieved statistical significance.

**TABLE 3:**
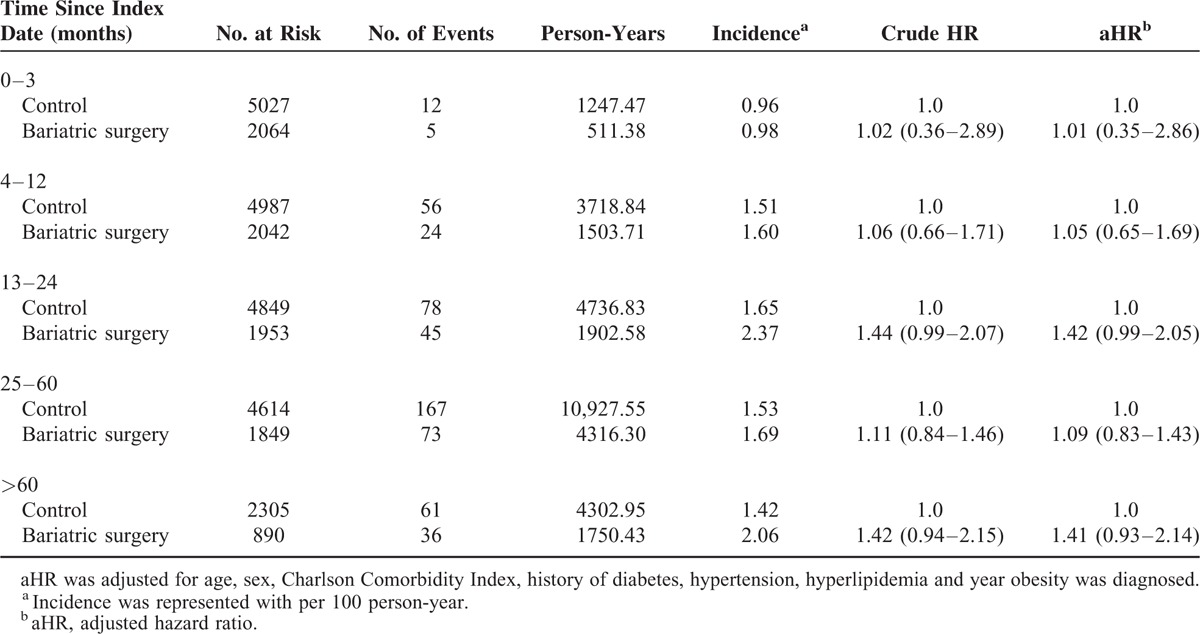
Risk of Any Fracture in the Patients Receiving Bariatric Surgery and the Matched Controls by Follow-Up Period

Table 4 shows the relative fracture risk in the surgical patients stratified by fracture site compared with the matched controls. Most fractures occurred in the 4 extremities (143 events, 78%) rather than in the axial skeleton. We also observed an increase in the overall risk of any fracture in the surgical group after adjustment (aHR: 1.21, 95% CI: 1.01–1.44); however, only atypical fracture sites, including the clavicle, scapula, sternum (aHR: 2.16, 95% CI: 1.27–3.68), and feet and toes (aHR: 1.53, 95% CI: 1.02–2.30) reached statistical significance. The adjusted HR of all lower extremity sites in surgical group was 1.29 (95% CI: 0.97–1.72) compared with matched controls. Furthermore, we stratified type of fracture into osteoporotic and non-osteoporotic fractures. Osteoporotic fracture included ICD-9CM 805 (fracture of vertebral column), 812 (fracture of humerus), 813 (fracture of radius and ulna), 814 (fracture of carpal bones), and 820 (fracture of neck of femur). Surgical group had a trend of increased risk of fracture compared with matched controls both in osteoporotic fracture (aHR: 1.05, 95% CI: 0.77–1.43) and nonosteoporotic fracture (aHR: 1.23, 95% CI: 0.99–1.50). (data not shown).

**TABLE 4:**
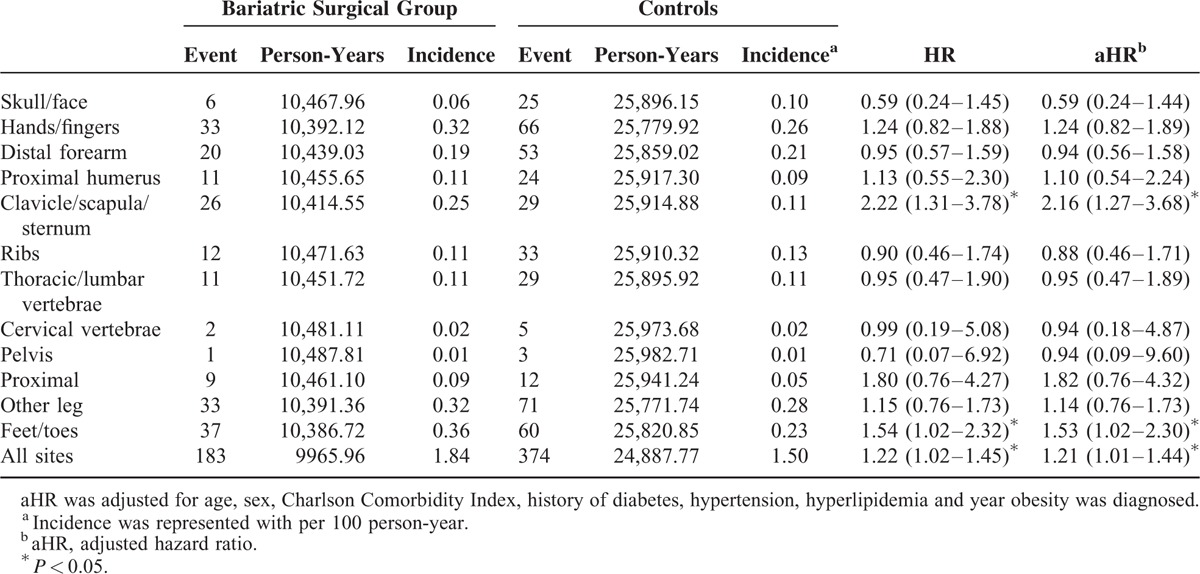
Risk of Fracture in the Patients Receiving Bariatric Surgery and the Matched Controls by Fracture Site

## DISCUSSION

To the best of our knowledge, this is the first study to delineate the risk of fractures in patients who underwent bariatric surgical procedures compared with matched controls in a nationwide, population-based cohort. We demonstrated that bariatric surgery, mainly with malabsorptive procedures, was significantly associated with an increased risk of overall fractures (aHR: 1.21, 95% CI: 1.02–1.43). This seems to be inconsistent with the results from a retrospective cohort in the UK, in which 2079 patients underwent malabsorptive or restrictive bariatric surgery, and no association with an increased fracture risk was found compared with the controls.^19^ In subgroup analysis, Lalmohamed et al, however, found a trend toward an increased risk of fracture in the group with greater reduction of excess body mass index (BMI) after surgery in this cohort. The mean follow-up time in the study was only 2.2 years, which may have reduced its statistical power. In addition, the medical records in the UK study came from general practice clinics, which only represented 8% of the population. That may have led to selection bias by excluding fracture events in secondary or tertiary services. In contrast, a study in Olmsted County in the United States demonstrated that bariatric surgery was associated with an increased fracture risk during 14 years of follow-up.^18^ Although it was a small-scale study, did not include matched controls, and did not adjust for well-known confounding factors such as chronic illnesses and comorbidities, the significant association prompted further investigation and clinical attention. The current study covered more than 99% of the residents in Taiwan, with a follow-up period of 12 years, and matched for age, sex, CCI, diabetes, hypertension, hyperlipidemia, and the year morbid obesity was diagnosed to eliminate possible confounding effects.

Weight loss per se is associated with decreased BMD. Bariatric surgeries with malabsorptive procedures were reported to result in higher weight loss than that with restrictive procedures.^8,9,24^ Furthermore, malabsorptive procedures have been linked with higher calcium and vitamin D deficiency, which is known to be important for bone health.^11^ In subgroup analysis, we found that malabsorptive procedures resulted in a significantly higher fracture risk (aHR: 1.47, 95% CI: 1.01–2.15) whereas restrictive procedures did not (1.17, 95% CI: 0.97–1.41).

A systemic review and meta-analysis suggested that bariatric surgery is associated with decreased BMD and therefore may indirectly increase fracture risk.^9^ These negative effects on bone health appeared to occur early, ranging from 9 months to 2 years, however, the effects over a sustained period were unclear. One prospective investigation reported that patients who underwent malabsorptive procedures had a decreased BMD 1 year later, but no significant changes after 3 years.^11^ A similar result was reported with restrictive procedures after 30 months of follow-up.^14^ In contrast, one study prospectively followed 37 patients who underwent malabsorptive procedures and found that BMD did not change after 4 years but significantly decreased 10 years later.^13^ We found that the fracture rates increased after bariatric surgery, and were 1.60% at 1 year, 2.37% at 2 years, 1.69% at 5 years, and 2.06% after 5 years. Adjusted analysis showed a trend (aHR: 1.42; 95% CI: 0.99–2.05) toward an increased fracture risk 1 to 2 years after bariatric surgery.

Although the mechanism behind the increased risk of fractures after bariatric surgery is not fully understood, estrogen and adipokines seem to be involved.^25^ Estrogen plays a fundamental role in bone homeostasis by inhibiting osteoclast and bone resorption.^26^ Adipose tissue is one of the main sites for estrogen production, and fat volume substantially decreases after bariatric surgery. One interventional study compared bariatric surgery and medical therapy between 2 obese groups, and found that estradiol level in the obese women was highly associated with bone loss in the surgical group.^27^ In addition, adipocytes secrete a wide variety of proteins, called adipokines, including leptin, adiponectin, and others which are known to be involved in bone physiology.^28^ Several studies have shown that leptin stimulates bone growth in vitro,^29^ increases bone density in animals,^30^ and significantly decreases after bariatric surgery.^31^ In light of these findings, a decrease in leptin after bariatric surgery may impair bone metabolism and then lead to a reduced BMD. In contrast, circulating adiponectin induces bone resorption and inhibits osteoblasts.^32^ This is supported by a prospective study, in which 42 obese women showed an increase in circulating adiponectin level 1 year after gastric bypass surgery.^24^ The opposite actions of decreased leptin and increased adiponectin may result in an increase in bone turnover marker, osteoclast recruitment and worse bone fragility after bariatric surgery.^33^

We also found that the majority of fractures after bariatric surgery occurred in the 4 extremities (143 events, 78%), which are not typical sites for osteoporotic fractures. In addition, only nonosteoporotic fracture sites (clavicle, scapula, sternum aHR: 2.16; 95% CI: 1.27–3.68; feet and toes: 1.53; CI: 1.02–2.30) were associated with an increased fracture risk. Prospective investigations, however, have reported evidence of decreased BMD in the hip region and, to a lesser extent, in the spine of women.^34^ Nakamura et al^18^ found an increased risk of fracture in surgical group at lower extremities, including proximal femur, other leg and feet/toes in Olmsted cohort. Similarly, there was an increase of fracture risk in lower extremities (aHR: 1.29; 95% CI: 0.97–1.72) without statistical significance in our study. In the UK study, there was no association between bariatric surgery and the risk of either osteoporotic or nonosteoporotic fractures.^19^ Our patients underwent bariatric surgery at a mean of age 31.8 years, which is at least 10 years younger than in Western countries.^3^ The mechanism behind this finding is unknown, however, we speculate that it may be more related to microarchitectural deterioration than quantitative T- and Z-scores. More and more investigation explored microarchitectural deterioration of bone after bariatric surgery by high-resolution peripheral quantitative computed tomography. Stein et al^35^ found that trabecular bone was stable, whereas cortical bone was deteriorated 12 months after bariatric surgery, particular in tibia area. Yu et al,^36^ however, followed 30 subjects underwent gastric bypass for 24 months and found that both cortical and trabecular bone were deteriorated at distal radius and tibia. We hypothesized that fractures may occur through increased bone turnover and altered bone fragility among cortical and trabecular bones, leading to poor bone strength rather than a greater loss of bone.

There are several limitations to this study. First and most importantly, our findings cannot address unobserved confounding factors, even though we performed propensity score matching to minimize impacts caused by the measured covariates. As a matched controlled cohort, the estimated HRs only demonstrated associations but not the causal effect of bariatric surgery on fractures. Besides, data on BMI presurgery and postsurgery were not available. Using ICD-9-CM diagnostic codes, we included all cases of morbid obesity defined as a BMI ≥ 40 kg/m^2^ or BMI ≥ 35 kg/m^2^ plus at least one comorbidity, however, we did not account for the severity of obesity. Furthermore, information on vertebral fractures without clinical symptoms and doctor visiting was not captured and this might result in an underestimate. In addition, we were unable to assess the use of self-administrated medications, which may have altered bone condition. Because the surgical patients were young (31.8 ± 9.2 years) and most did not have any underlying diseases (77.8% of the surgical patients had a CCI score of 0), we speculate that the use of bisphosphonates and hormone replacement therapy accounted for a very small proportion of the study group. Similarly, there was no data on vitamin D, calcium, and parathyroid hormone. We did not record vitamin D deficiency or hyperparathyroidism, which might include subjects with secondary osteoporosis or postoperative malabsorption. Besides, an optimized multivitamin supplement has been demonstrated to reduce the development of some nutritional deficiencies after bypass surgery.^37^

Understanding the long-term outcomes of bariatric surgery on bone health is a priority for public health worldwide. In this nationwide cohort, bariatric surgery was significantly associated with an increased risk of fractures. These results provide further evidence for the adverse effects of bariatric surgery on the risk of fractures.
